# Association for the Oral Instruction of the Deaf and Dumb

**Published:** 1903-06-20

**Authors:** 


### ASSOCIATION FOR THE ORAL INSTRUCTION OF THE DEAF AND DUMB.

Earl Carrington presided at a dinner in aid of the funds of the Association for the Oral Instruction of the Deaf and Dumb, at the Hotel Cecil, on Thursday, June 11th.

After the usual loyal toasts had been duly honoured, the Chairman rose to propose the toast of the evening, but before doing so desired to give expression to the regret he was sure they all felt at the absence of the hon. secretary, Mr. Assur Moses, through a most severe domestic affliction—the death of one of his sons. Proceeding, he said the Association was founded in 1870, and had its origin in the kind heart and thought of the late Baroness Mayer de Rothschild. It was originally intended for children of the Jewish faith, but owing to the great success of the method of teaching, its scope was extended to poor children of all classes and creeds. It had been carried on on purely commercial lines, and they had only had three festival dinners in the three and thirty years since its foundation. The first, in 1877, was presided over by the Prince of Wales, now the King; the Lord Mayor presided in 1890, and the Duke of York, as he then was, in 1897, when the Queen and the Princess of Wales showed their interest by attending a public examination of the children. In 1886 a Royal Commission ordained that the pure oral system should be carried out in all public institutions. The system was fast becoming the national one, and the Association now supplied teachers to the London School Board, to the Colonies, and to India. While congratulating the Association on the fact of having had only three dinners in 33 years, yet he must remind his hearers that the institution had no endowment or property except a small freehold house, while it had a small debt at its bankers. He had visited the house in Fitzroy Square, and it was quite evident it was in want of repair and particularly of a new workshop on an upper story. The working expenses amounted to £500 a year, which must be procured somehow. The directors had, therefore, wisely determined to hold this their fourth dinner in their thirty-fourth year, and he was informed that they wanted £5,000 to keep their heads above water. When he paid his visit he did not know what he was going to see, but he came away much wiser than when he went. He saw there what he could not have believed possible. They all knew the difficulty of making deaf people understand, and how they felt cut off from society. That was hard for grown-up people, but specially so for children. Yet these little children he saw, three, four, and five years old, born deaf, and yet, though unable to hear were able to understand and to make sounds that meant words. By the patience of good women these children were trained so that they were able to keep up animated conversations and learn difficult trades. He did from the bottom of his heart recommend this charity to that gathering, and through them to the world at large, so that its benefits might be more widely extended. He urged them not only to subscribe liberally, but to get their friends to give their support also. In conclusion he would quote the divine precept: "In as much as ye have done it unto one of these little ones, ye have done it unto Me."

Mr. Van Praag then read out the list of donations, which amounted to £3,400, the announcement being received with hearty applause. He hoped, he said, that before leaving the room the sum would be made up to £5,000; but if any gentleman was unable to remain long enough to be persuaded to that extent, if he would only come round to No. 11 Fitzroy Square, he should be happy to complete his conversion.

Toasts to the stewards, the committee and executive, and the chairman followed.

